# An automated cell-tracking pipeline for the analysis of neutrophil dynamics

**DOI:** 10.3389/fbinf.2026.1748364

**Published:** 2026-04-21

**Authors:** Chen Li, Wilson W. C. Yiu, Wanbin Hu, Herman P. Spaink, Lu Cao, Fons J. Verbeek

**Affiliations:** 1 Leiden Institute of Advanced Computer Science, Leiden University, Leiden, Netherlands; 2 Institute of Biology, Leiden University, Leiden, Netherlands

**Keywords:** cell tracking, neutrophil migration, U-Net, Viterbi algorithm, zebrafish

## Abstract

Neutrophils play a key role in the innate immune system. They act as the primary line of defense when bacteria, viruses, or other harmful foreign particles invade the immune system. Accurate movement measurement of neutrophils, including velocity, direction, and displacement, is crucial to studying the regulation of cell migration behavior. Cell tracking is a key technology to realize the quantification of these measurements. In this article, we developed a pipeline, including cell segmentation, cell motion tracking between two frames, and trajectory linkage, to realize cell tracking. Our starting point was to collect time-lapse sequences of neutrophils using a confocal microscope. We pre-processed each frame in the time-lapse sequence to improve the image quality by denoising, smoothing, and contrast enhancement. Subsequently, a deep learning model, that is, U-Net, was used to segment cells in each image frame. U-Net was used again to track the cells between two adjacent frames by calculating the score matrices representing the posterior probability of linkage. Moreover, an extended Viterbi algorithm was applied to find optimal trajectories based on score matrices generated by the U-Net. Results demonstrate that our pipeline outperforms other representative linkage methods used in cell tracking. It provides a robust, practical solution for a challenging and highly motile *in vivo* regime.

## 1 Introduction

Acute inflammation could be identified by the monitoring of neutrophil migration, which can be caused by invading pathogens (Hu et al., 2021). Neutrophils are crucial immune cells. The migration pattern of neutrophils reflects the process *in vivo* of the innate defense mechanism against invading pathogens. However, the underlying mechanisms of neutrophil migration are not yet completely understood. In the field of biology and medicine (Hayashida and Bise, 2019; Ullah et al., 2016), microscopy techniques enable observing cells at high spatial and temporal resolution and advance the development of the dynamics analysis of immune cells. As the first line of defense against harmful pathogens, the exploration of movements of living neutrophils, *in vivo*, in the spatial–temporal domain can contribute to a better understanding of their behaviors and mechanisms.

Cell tracking is a key technology for quantitative analysis of neutrophil migration. It aims to identify the trajectory of each cell in a time-lapse sequence. However, due to the complexity of cell behavior, this field still lacks accurate and automated algorithms. The manual tracking of cells is a time-consuming and challenging task. Particularly, with the introduction of high-throughput screening, it becomes impossible to conduct manual tracking for a large number of image datasets. In addition, cell motility can systematically distort time-lapse measurements if cell identity is not preserved correctly over time. In particular, prior work on cytometry of reaction rate constants (CRRCs) explicitly shows that cell movement during time-lapse imaging can invalidate fixed-contour measurements and that accurate cell tracking is required to extract reliable single-cell kinetic parameters (Koshkin et al., 2019; Nebbioso et al., 2023). The original formulation of CRRCs quantifies heterogeneity in single-cell reaction rates. Its later extension shows that accurate contour tracking is essential when cells are motile. Improved tracking directly affects distributions of inferred kinetic constants rather than merely improving visualization. These studies stress that tracking improvements is not cosmetic but can fundamentally change quantitative conclusions. Therefore, developing automatic and robust cell-tracking approaches is essential.

In biomedical research, cell tracking is generally preceded by cell detection and tracking, which is based on either single-cell tracking or multi-cell tracking (Hernandez et al., 2018; Scherr et al., 2020; Yan et al., 2009; Lugagne et al., 2020). The idea of tracking by detection is to segment cells from each frame first and then associate the cells frame by frame. Cell segmentation algorithms are designed to localize the single-cell object in each frame. The existing approaches are based on different strategies such as thresholding, watershed, and deep learning neural networks (Arbelle et al., 2018; Al-Kofahi et al., 2018; Vicar et al., 2019; Xing and Yang, 2016). The Otsu adaptive thresholding method (Nobuyuki, 1979) is one thresholding strategy that can find an optimal value by maximizing the variance between foreground and background and distinguishing them.

The watershed algorithm described by Vincent (Luc and Pierre, 1991) calculates the gradient magnitude of the image to identify potential region boundaries, and markers are selected and placed on the image. The watershed transform simulates a flooding process, during which water is poured into the valleys, gradually filling them up. When water from different regions meets at the same point, dams are formed, which represent segment boundaries. This guide is for partitioning an image into distinct regions or objects.

These rule-based approaches are reliant on human intervention for fine-tuning parameters. Recently, supervised deep learning methods have become popular due to their learning-based capacity and superior results in the field of cell segmentation. U-Net (Ronneberger et al., 2015) is a successful deep learning model for biomedical image segmentation because of its light-weight and well-performed architecture. Subsequently, different improved versions of U-Net, such as Res-UNet (Xiao et al., 2018) and Dense-UNet (Guan et al., 2020), have been constructed and applied on biomedical segmentation tasks. U-Net is originally a semantic segmentation method that focuses on the separation between foreground and background. It could not identify each object individually if objects are colliding or overlapping. Cell segmentation, as a pre-step of tracking, is expected to separate every single cell from the image. This can be achieved by instance segmentation. A network with one encoder and two decoder paths was designed based on the U-Net structure, along with a watershed post-processing, to perform the instance cell segmentation (Scherr et al., 2020). It has achieved top performance in the IEEE ISBI 2020 Cell Tracking Challenge. All these related studies confirm the power of the U-Net architecture for biomedical image segmentation tasks.

Once cell segmentation is performed, the next step is cell association. It determines the linkages between cells on a frame-by-frame basis. Two classic linkage methods are the nearest-neighbor method and linear programming algorithms (Xing and Yang, 2016; He et al., 2017). The nearest-neighbor method links the cells to the nearest cell on adjacent frames based on the characteristics of cells such as intensity, shape (Turetken et al., 2017), and motion. For instance, Yan et al. (2009) proposed an algorithm named Kernel Density Estimation Mean Shift (KDE) as a linkage solution. It started by converting the initial object into density models and recursively updated the mean shift factor based on a local density in consecutive frames until a stationary location is reached. The cell closest to the stationary point was chosen as the candidate. However, a simple nearest-neighbor linkage method could not solve a more complex cell behavior well—that is, events such as cell appearing, disappearing, merging, and splitting during the movements. Linear programming algorithms successfully solved these problems (Hernandez et al., 2018; Xiao et al., 2018; He et al., 2017). The Viterbi algorithm is a classic linear programming algorithm. It is a global method to link cell trajectories based on the probabilistic functions or scoring functions obtained from feature similarities of cells. Excellent performance of the Viterbi linkage algorithm has been reported (Hernandez et al., 2018; Magnusson and Joakim, 2012; Magnusson et al., 2015). In summary, cell association methods need handcrafted cell similarities to link cells between frames.

In recent years, deep learning methods have become popular for cell-tracking tasks. They eliminate the step of manual feature extraction for cell similarities (Lugagne et al., 2020; Payer et al., 2019; Moghadam and Chen, 2021; Moen et al., 2019). There are two ways to realize cell tracking using deep learning methods. One is a two-step approach that a deep learning model learns the cell migration patterns from the ground-truth data, which replaces the process of handcrafted cell similarity extraction. Subsequently, a linkage method is constructed. Lugagne et al. (2020) proposed a U-Net structure for learning the cell movement patterns from an *E. coli* dataset. From the predictions of the tracking model, a set of score matrices, including the probability of each pair of cells among all consecutive frames, was calculated. A nearest-neighbor linkage method was used to associate the cells over the frames, by which a sound performance was achieved.

Another method is a one-off training process. A deep learning model is trained to simultaneously learn the cell movement patterns and the linkages. Payer et al. (2019) presented a novel recurrent stacked hourglass network for instance segmentation and tracking. They reached state-of-the-art performance on their own dataset of muscle fibers and six datasets from the ISBI Cell Tracking Challenge. The network was trained on a dataset including both segmentation and tracking ground-truth data for each frame. The network used cosine embedding loss for smoother embeddings within instances and faster network convergence. However, their evaluation datasets do not allow a cell to disappear and recur later in time.

Moghadam and Chen (2021) proposed a cell-tracking framework based on a novel multi-channel feature learning model. This model can learn the spatial feature from convolutional layers and the temporal feature from the center relocation distance layer and the orientation layer simultaneously over time-lapse sequences from the ground-truth data. They built a ground-truth dataset by manually cropping individual cells of any consecutive frames to form image pairs. The pairs of images were annotated as true if they were the same cell in two adjacent frames; otherwise, they were annotated as false. We observed that simultaneous cell tracking in spatial and temporal feature space with a deep learning framework is gradually becoming a trend. However, it still poses several limitations. One limitation is that there is no standardized procedure for the preparation of ground-truth data used in simultaneous cell tracking models. In addition, it is very challenging and laborious to prepare ground-truth data. This is especially the case for the complex time-lapse sequences in our research, such as neutrophil movement. Therefore, a two-step tracking strategy is most commonly used in the existing literature and is also preferred for our study.

Recently, many standalone tools have been developed in support of cell-tracking tasks, such as TrackMate (Tinevez et al., 2017), PhagoSight (Henry et al., 2013), TrackPad (Cornwell et al., 2020), CellProfiler (Carpenter et al., 2006). In general, these open-source and interactive tools integrate many functionalities, including cell segmentation, tracking, visualization, manual correction, and analysis. These tools have advanced the field of cell tracking for different cell types; however, they do not fit all types. Complex cell events are encountered in our neutrophil dataset, such as appearing, disappearing, merging, and splitting. Existing tools have difficulty tracking these cells accurately.

Based on these motivations, we designed a cell-tracking pipeline to track the complex migration dynamics of neutrophils for our research. The proposed pipeline comprises three steps. For the first step of cell segmentation, different segmentation models were investigated. The U-Net model was recognized as the most suitable model to segment neutrophils from images. Subsequently, a two-step association strategy was applied. The scoring function of cell similarities was obtained by another U-Net-based tracking model. It learned the migration patterns between adjacent frames from the ground-truth data. Finally, an extended Viterbi linkage algorithm was proposed to solve the complex linkage problem. Compared with the other state-of-the-art methods (Yan et al., 2009; Lugagne et al., 2020; Atsuki et al., 2015), our algorithm achieved the lowest mean value of 0.010 for falsely identified tracker (*FIT*) and 0.173 for falsely identified object (*FIO*), together with the highest track purity (*TP*) value of 0.763 and a second-best object purity (*OP*) of 0.299 for our neutrophil dataset.

The contributions of our research are summarized as follows: (1) We built a ground-truth dataset of neutrophil migration for the zebrafish model, which provides a potential chance for other researchers to investigate similar programs; (2) we developed a pipeline to solve the neutrophil tracking challenges, which integrates cell segmentation, cell motion tracking, and trajectory linkage; and (3) we designed a creative Viterbi algorithm for trajectory linkage. Different heuristics were formulated for specific migration patterns of neutrophils, which aim to solve complex tracking problems.

## 2 Materials and methods

### 2.1 Biological role of each step in the workflow

**FIGURE 1:**
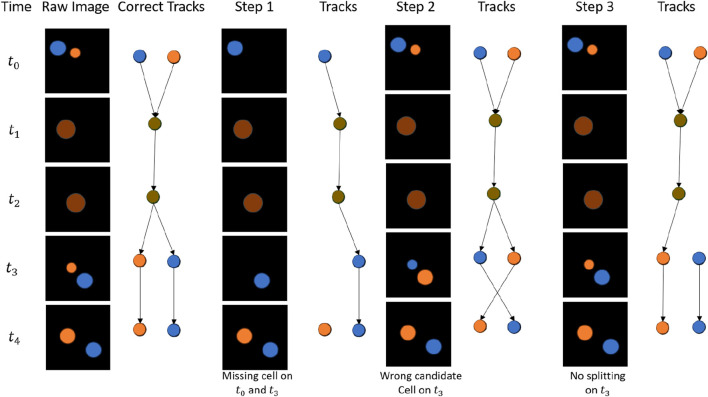
Potential errors in different steps (Step 1: cell identification; Step 2: candidate cell prediction; and Step 3: trajectory linkage).

In general, our cell-tracking workflow consists of cell identification, candidate cell prediction in the next frame, and cell trajectory linkage. Each step is optimized to prevent different types of tracking errors. At the first step of cell identification, small cells or cells with low fluorescent signals could be missed. Temporarily overlapping cells would be segmented as one merged cell. In addition, the segmentation method could wrongly split one object into multiple cells (over-segmentation). Missing cells cause wrongly linked or missed trajectories. Merged cells force the design of new strategies to incorporate merging and splitting in the linkage stage. Overly split cells erroneously create more splitting trajectories. At the second step of predicting the candidate cell in the next frame, the wrong candidate cell or more than one candidate cell could be predicted. It causes the wrong linkage or a new trajectory for an incorrectly predicted splitting scenario. At the last step of trajectory linkage, an error occurs when the cells are merging, splitting, and moving in and out of the view. If these situations are not treated properly, the created trajectories would be wrongly linked, incomplete, or unnecessarily long. We include a simplified comparison showing how tracking results change when individual steps are not optimized in Figure 1.

**FIGURE 2:**
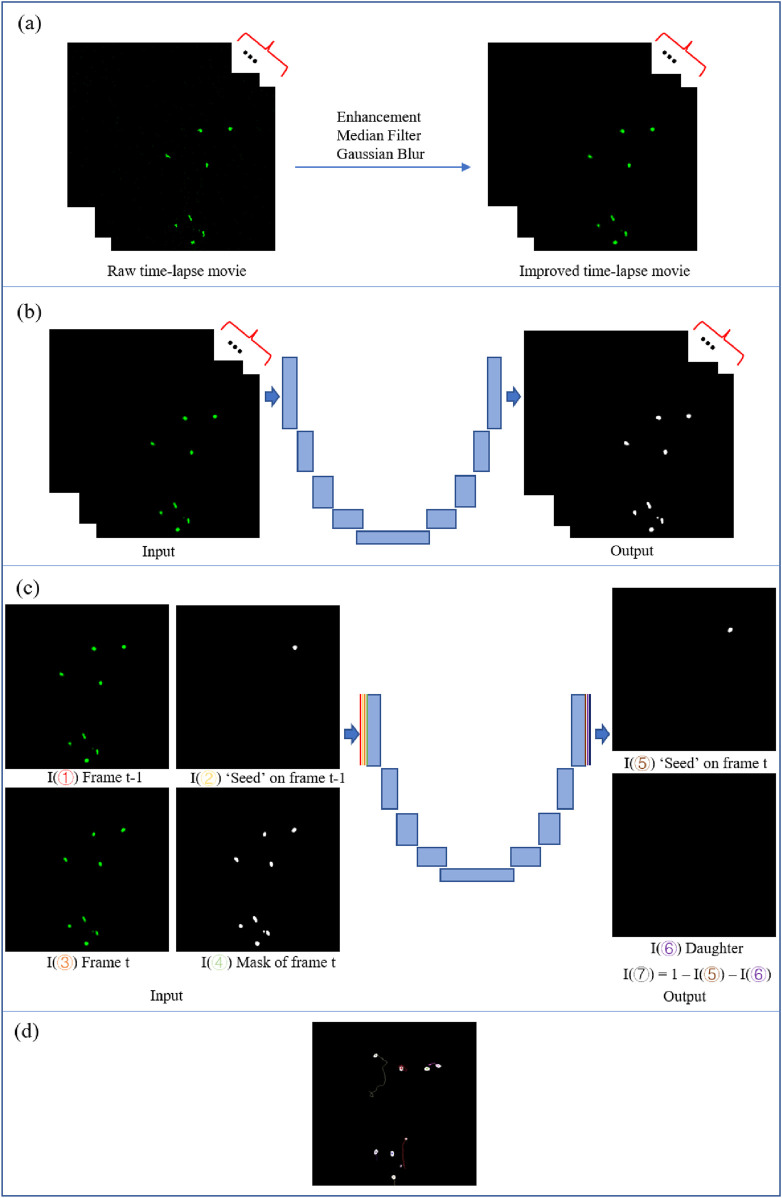
Pipeline of cell tracking. **(a)** Data pre-processing. **(b)** U-Net segmentation model. **(c)** U-Net tracking model. I(①)I(②)I(③)I(④) represent the four inputs, and I(⑤)I(⑥)I(⑦) represent the three outputs of the model. I(⑦) is calculated by the inversion of the other two outputs, I(⑤) and I(⑥). **(d)** Viterbi linkage and visualization.

The detailed framework of our pipeline is shown in Figure 2. Pre-processing was done to improve the image quality of the time-lapse sequence. Two U-Net-based deep learning models were applied, respectively, for cell identification/segmentation and tracking. After training the tracking model, the predictions of the possible cell location on the next frame were generated. Finally, the extended Viterbi algorithm was performed to link the trajectories based on the predictions. The trajectories were visualized through a time-lapse sequence. More methodology details are introduced in the following subsections.

### 2.2 Image capturing and pre-processing

The neutrophil time-lapse sequences are captured from the tail of a zebrafish after an induced tail-wounding. All the experiments with zebrafish followed the international guidelines specified by the EU Animal Protection Directive 2010/63/EU. To quantify leukocyte responses at the wound edge, we used Tg(mpeg1:mCherry-F); TgBAC(mpx:EGFP) zebrafish (*Danio rerio*) larvae in tlr2 or myd88 mutant backgrounds and the corresponding wild-type controls. Time-lapse imaging was performed on 3-day-post-fertilization (dpf) larvae from 1 hour-post-wounding (hpw) to 3 hpw. Larvae were immobilized in 0.02% tricaine and mounted in 1% low-melting-point agarose (Sigma-Aldrich) (Hu et al., 2021). Imaging was carried out on a Leica TCS SP8 confocal laser scanning microscope (CLSM) using a 20 × objective (NA 0.75) at 1-minute intervals for 2 h, with samples maintained at 28.5°C . Time-lapse images were acquired with z-stacks. To minimize phototoxicity, imaging conditions were kept consistent across groups and restricted to the lowest excitation/exposure compatible with reliable cell detection.

We carried out preliminary tests before finalizing the imaging settings. Because we were interested in measuring neutrophil migration properties after cell tracking, such as total displacement, we aimed to use the shortest possible time interval. In addition, we imaged the tail fin of zebrafish larvae, which is a very thin tissue containing only two cell layers. Therefore, we imaged an 8-layer 3D stack. Additionally, we had a limitation on the scanning area because we needed to image at least two fish at the same time (one from the wild-type and one from the mutation group). Thus, a 1 min interval was the shortest time interval we could use. Neutrophils localized within an area of 200 µm from the wounding edge toward the body trunk were counted as recruited cells. The zebrafish tails were scanned from top to bottom under the microscope. An 8-layer 3D stack at each time point was captured. The image stack size is 512 × 512 × 8. The layer interval is 5–6 µm, while the thickness of the tail is 35–42 µm. For each sample, a 2-hour time-lapse sequence was captured. There are 120 frames for each time-lapse sequence. Each time-lapse sequence contains around 10–40 cells. We captured 20 time-lapse sequences in total.

**FIGURE 3:**
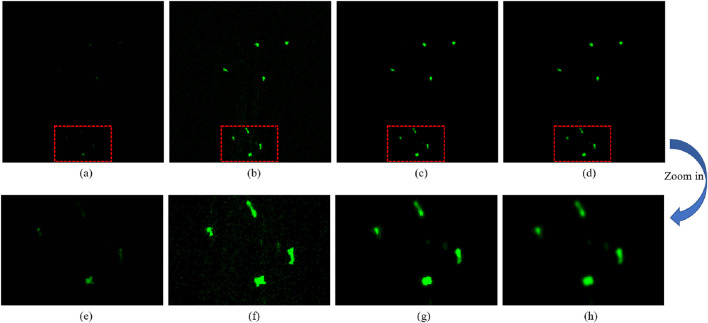
Data pre-processing. **(a)** One frame of the raw data. **(b)** Image contrast enhancement. **(c)** Image denoising using a median filter. **(d)** Image smoothed using a Gaussian blur. **(e–h)** Corresponding zoomed-in areas of the red boxes in **(a–d)**, respectively.

The raw data we obtained are thus 3D + t time-lapse sequences. Due to the thin tissue layer of the fish fin and relative undersampling in the Z-axis direction, for this article, we intend to focus on solving the tracking problem for neutrophils in 2D space and to evaluate how well a 2D cell-tracking method can solve the problem. To that end, a maximum intensity projection was applied to the 8-layer image stack to derive 2D projection data at each time point. In Figure 3a, the first projected frame of one sequence is depicted. The intensity is relatively low, and the cells are not clearly visible. Therefore, image pre-processing steps were applied to improve the image quality. First, we enhanced the image contrast to highlight the cell; cf. Figure 3b. However, this also increased the background noise. Therefore, a median filter was used to denoise; cf. Figure 3c. Subsequently, we chose a Gaussian blur to smooth the cell surface area; cf. Figure 3d. The corresponding magnified areas in the red boxes of Figures 3a–d are shown in Figures 3e–h, respectively, to clearly present the image details.

### 2.3 Cell segmentation

#### 2.3.1 Ground-truth labeling for segmentation

Annotated images are used for the stages of both training and evaluation of segmentation models. We used AnnotatorJ (Hollandi et al., 2020) to label each cell. This is a semiautomatic tool to find an approximate cell mask by applying U-Net-based pre-segmentation. It follows a manual correction to precisely create the masks of individual cells. Users can create their own annotated dataset based on different options: binary mask annotation and instance mask annotation. In our study, we created 240 images of instance mask annotations. Binary mask annotations can be obtained by thresholding. Both binary and instance mask annotations were used to train the U-Net semantic segmentation model and instance segmentation model, respectively, and to evaluate the segmentation performance.

#### 2.3.2 Segmentation models

A good segmentation model is expected to identify each cell, without missing cells, over-segmentation, or under-segmentation. In our study, the fluorescence imaging condition is stable. Neutrophils are morphologically irregular but are mostly well separated in frames. These conditions allow a light-weighted U-Net to achieve accurate segmentation that is suitable for cell tracking. As a result, we prioritized comparison among the U-net-based models, including two variants for semantic segmentation and one variant for instance segmentation.

First, we implemented a U-Net model (U-Net 1). It has a similar structure to the original U-Net model (Ronneberger et al., 2015) but without an overlap-tile strategy. Both the input and output sizes of the network were set to 512 × 512, which is the same as the raw image size. U-Net 1 was trained over 10 epochs of 300 steps when the performance was saturated. It was trained on the Tensorflow platform on a dedicated server equipped with two NVidia GeForce GTX 2070/8 GB GPUs. The batch size was set to 1 due to the large image size and the limitation of GPU memory. Data augmentation was applied to increase the number of training sets and avoid overfitting of the network. In our case, width and height shift and vertical and horizontal flip were used to augment our dataset because these transforms do not change the cell morphology. For our dataset, the pixel number of the foreground (positive sample) and the background (negative sample) is unbalanced. The negative samples occupy the largest area of the image. The consequence is that no candidate cells would be predicted and generated by the network. Thus, selecting a suitable loss function plays a key role during training. Dice loss function and focal loss function (Yeung et al., 2021; Sudre et al., 2017) have been demonstrated to be very effective in solving class unbalanced problems and thus were compared in our experiments. Subsequently, the original U-Net model (U-Net 2) (Ronneberger et al., 2015) was implemented. We intended to compare both nets. The differences between U-Net 1 and U-Net 2 are as follows: U-Net 2 works with an overlap-tile strategy. The input and output sizes are 512 × 512 and 388 × 388, respectively. The output images must be resized to 512 × 512 to obtain the same size images as our raw data.

In addition, an instance U-Net segmentation model (U-Net 3) (Scherr et al., 2020) was adopted. The structure of U-Net 3 has one encoder path and two decoder paths, which aim to perform an instance-based segmentation task. This model was used to test the possibility of using an instance-based segmentation model to solve the under-segmentation problems encountered in the two U-Net-based semantic segmentation models. In addition to the deep learning models, a rule-based watershed masked clustering (WMC) segmentation algorithm (Yan and Verbeek, 2012) was included because it has been used with a similar cell-tracking task.

#### 2.3.3 Evaluations for segmentation

To evaluate the performance of the segmentation models, we collected five commonly used evaluation metrics (Caicedo et al., 2019). The first one is the Jaccard index, which is defined as follows:

$$IoU\left(T,E\right)=\frac{\left|T\cap E\right|}{\left|T\cup E\right|}.$$ (1)

Here, T represents the ground-truth image, and E represents the predicted segmentation image. The metric relies on the computation of intersection-over-union (*IoU*) between a pair of objects on T and E , respectively. *IoU* measures the pixel-wise foreground overlap between them.

The second commonly used evaluation metric is the *F1* score. We measured the number of true positive (*TP*), false negative (*FN*), and false positive (*FP*) cells. *F1* score is computed as follows:

$$F1=\frac{2TruePos}{2TP+FP+FN},$$ (2)

where *TruePos* represents the corresponding pair of objects on both T and E . *FP* represents the objects that are predicted on E while not annotated on T . *FN* is the objects that are missed by the segmentation model compared with T .

*FN* is considered a third evaluation criterion individually. Because a missing cell is not expected in the tracking task, it causes a loss of target and a wrong connection of the trajectory. In addition to *IoU*, *F1* score, and *FN*, we also counted the number of under-segmented and over-segmented cells. These two measurements reflect the ability of the segmentation model to solve the under-segmentation and over-segmentation problems.

In principle, a better performance is achieved with a higher *IoU* and *F1* score, together with a lower cell number of *FN*, under-segmentation, and over-segmentation. In practice, however, all these metrics are interrelated. They depend on the value of threshold t that sets the predicted probability of each pixel above t as foreground and below t as background. If t is too high, the number of pixels that are recognized as the foreground decreases. This causes the predicted cells to have a much smaller surface area, resulting in a lower *IoU* value, a higher number of over-segmented cells, and a lower number of under-segmented cells. In addition, some small cells were missed, which caused *FN* to increase further. Similarly, if t is too low, we obtain contrary results. In our experiment, we set t=0.1 to keep a balance among all evaluations.

### 2.4 Cell tracking

#### 2.4.1 Ground-truth labeling for tracking

For a supervised deep learning model for tracking, the ground-truth dataset is required. To obtain such a dataset, manual tracking was performed by experts (Hu et al., 2021) using the ManualTrack plugin (Meijering et al., 2012; Torraca et al., 2015). For each time-lapse sequence, the expert labeled three to nine trajectories. In total, there are 110 trajectories from all 20 time-lapse sequences. The (x,y) coordinates of each trajectory were recorded, which were used to localize the cells’ positions in the time-lapse sequence.

#### 2.4.2 U-net for tracking and evaluations

A U-Net-based deep learning model was adopted to learn the features of cell motion from adjacent frames of the ground-truth trajectories.

The U-Net-based model was designed for tracking *E. coli* cells trapped in mother machine microfluidic chambers (Lugagne et al., 2020). In this case, the cell movements were constrained in the vertical direction. It is a simplified cell-tracking problem. We intend to apply and extend this idea to a more complicated case. The U-Net architecture has four inputs and three outputs (cf. Figure 2c). For every cell at every time point of the ground-truth trajectory, there are four inputs and three outputs. The four inputs include the current frame, the segmentation mask of the current frame, the previous frame, and the “seed” cell that we want to track from the previous frame. The three outputs include the seed cell on the current frame, the potential daughter cell on the current frame if a division just happened, and the mask image of the reversion of the other two output images. For our dataset, we generated the training set, including input and output images from ground-truth trajectories based on the recorded coordinates. Cell division events do not occur in our case; thus, all the daughter images are empty.

In this study, we divided all 110 ground-truth trajectories into training, validation, and test sets based on a ratio of 8:1:1. We compared the performance of three tracking models on the test set. The first one was the pretrained model, which was already trained on yeast cells (Lugagne et al., 2020). This model was trained over 400 epochs of 250 steps per epoch with a batch size of 5 and a learning rate of 1× $10^{-4}$ . We used this model to evaluate the performance of the test set directly. We trained the other two models using TensorFlow on GPUs. One concerned a model built from scratch. All the parameters of the network are randomly initialized, and we trained this model based on our neutrophil training set. This model was trained for 20 epochs with mini-batches of size 1, 250 steps per epoch, and a learning rate of 1× $10^{-5}$ . The other model concerned a fine-tuned model. We fine-tuned the pretrained model on our training set to update the parameters of the network. This network was trained for 20 epochs, when the performance was saturated, with a batch size of 1 and a learning rate of 1× $10^{-5}$ . These hyperparameters were set based on experimental fine-tuning and to be consistent between the two trained models.

After we trained these three U-Net models, we evaluated the performance of each model by counting the number of incorrectly predicted cells on the test set.

#### 2.4.3 Extended Viterbi linkage method

**FIGURE 4:**
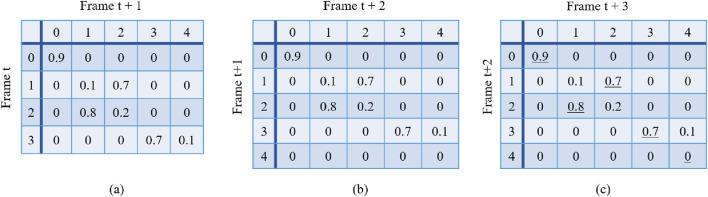
Score matrices. **(a)** Matrix between frame t and t+1 . There are four cells in frame t and five cells in frame t+1 . **(b)** Matrix between frame t+1 and t+2 . **(c)** Matrix between frame t+2 and t+3 . Each value in the matrix represents the computed score of each pair of cells on adjacent frames.

After the training, the images of predictions were generated from the tracking models. The score matrices were computed from these predictions. The pixels attributed to the cell in each predicted image were matched to the pixels in the segmentation mask of the corresponding frame. The overlap area of each pair of cells on the two images was calculated, and a score matrix for this specific time point was generated. Figure 4 shows three simple examples of score matrices among four consecutive frames. The number of rows represents the number of cells in frame t , and the number of columns represents the number of cells in frame t+1 . The values in the matrix represent the score between each pair of cells of the two adjacent frames. Each time-lapse sequence in our study has 120 frames in total. Therefore, we can obtain 119 adjacent score matrices for each time-lapse sequence.

Based on these score matrices, our aim was to find the optimal path for each tracked cell that achieved the highest accumulated score over the whole time-lapse sequence. A linkage method was required subsequently.

**FIGURE 5:**
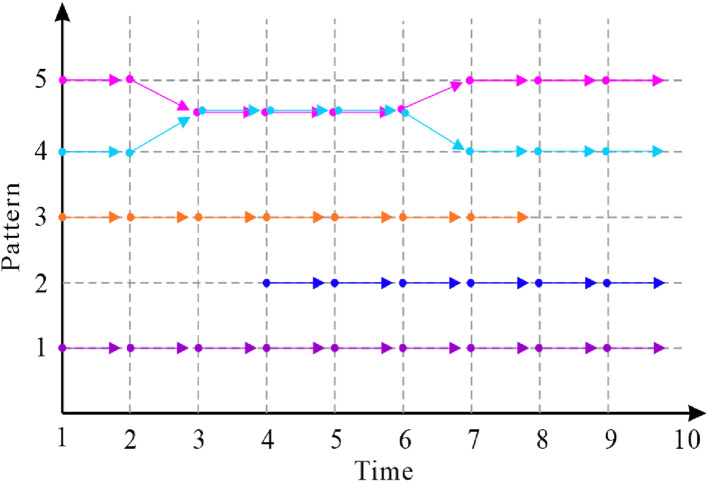
Cell behavioral patterns in neutrophil time-lapse sequences. Pattern 1: migration over all frames; Pattern 2: newly appearing; Pattern 3: disappearing; and Patterns 4 and 5: merge and split.

**FIGURE 6:**
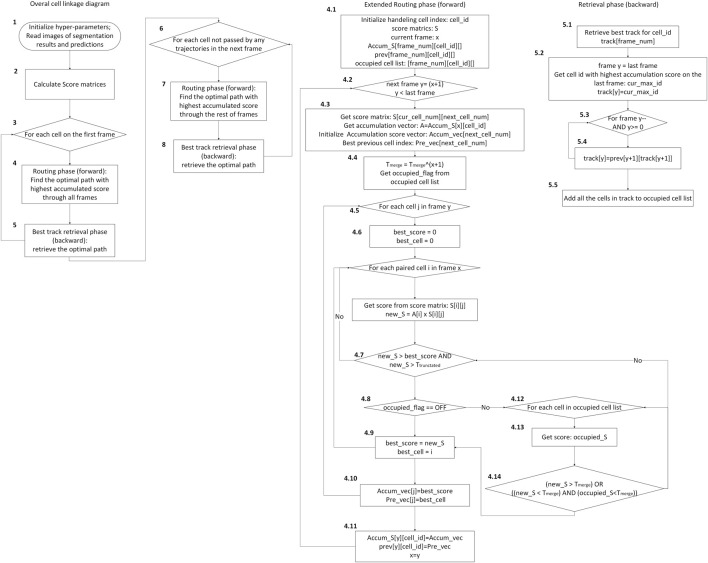
Flowchart of cell linkage algorithm, including the extended routing and retrieval phases.

Before introducing the linkage method, identifying the possible behavioral patterns of neutrophils can help us design a customized strategy for each pattern. We have observed and identified the following patterns of neutrophil movement: (pattern 1) migration over all frames in the sequence; (pattern 2) newly appearing cells; (pattern 3) disappearing cells; and (patterns 4 and 5) merging and splitting of cells. These patterns are depicted in Figure 5. For our 120-frame time-lapse sequence, very few neutrophils are always in the field of view of the images (pattern 1). In most cases, cells go in/out of view at some time point, which are defined as appearing and disappearing of cells. Merge and split patterns are the key problems that we aim to solve with additional strategies. In this study, an extended Viterbi linkage method was proposed. The use of the Viterbi algorithm was motivated by the power of the global track linkage and its superior performance in muscle stem cell (MuSC) and myoblast cell-tracking problems in Magnusson et al. (2015). The optimal trajectory for each cell is ensured by solving a dynamic programming problem over a state space diagram. The whole cell linkage diagram is shown in Figure 6. We incorporated the basic two-phase logic of the Viterbi algorithm: a routing phase (forward) at steps 4 and 7, and a best track retrieval phase (backward) at steps 5 and 8. Step 4 routing phase starts from each cell on the first frame, iteratively, to ensure the optimal path based on the accumulated score on each node of the state space diagram. It is detailed in steps 4.1 to 4.11. After all the cell trajectories in frame 1 were found, the cells that did not pass by any of those trajectories in the next frame were treated as the newly appearing cells at step 6. For the disappearing cell, the corresponding value in the score matrix between itself and all the candidate cells in the next frame is very low. Here, we set a truncated threshold $T_{\mathrm{truncated}}$ . The score of a cell that is lower than $T_{\mathrm{truncated}}$ used at step 4.7 was treated as a disappeared cell, referred to as the cell out of the field of view.

One drawback of the basic Viterbi algorithm is that it cannot deal with merge/split problems. Once the cell trajectories merged due to the collision or occlusion of cells at the same time point, the trajectories never split because they shared the same score matrices after the merge happened and ended up with the same optimal trajectory. This circumstance occurs as the Viterbi algorithm treats each track as independent. However, the calculation of an individual cell trajectory is largely affected by the surrounding tracks. Therefore, to solve this problem, an extended Viterbi algorithm, with extra steps 4.12 to 4.14, was developed to incorporate the surrounding tracks. To that end, an alternative path routing is developed on top of the basic Viterbi algorithm to manage the merge and split scenarios among cell trajectories. In the routing phase, the best node to frame t−1 could be occupied by one or more cell tracks. In such circumstances, connection to that node would only be allowed if the following conditions were satisfied:

Condition 1: The total score of the routing track is above the threshold T_merge up to that layer, or
Condition 2: All occupied cell track scores on the specific node are below the threshold T_merge up to that layer.

The threshold T_merge is created to support the alternative path routing. It provides a reference for merge/split decisions.

The initial value of the threshold is an adjustable hyperparameter. The threshold value must be discounted for every additional frame because the connection score has a decreasing trend from probability multiplication when routing to each new frame in step 4.4. The discount rate is calculated using the following equation.

$$DiscountThreshold={T_{\mathrm{merge}}}^{frame\text{\_}number-1}.$$ (3)

In addition, a reroute strategy is constructed. During the process of routing, a circumstance occurs when the later-routed track is a much better match than an occupied node in an earlier track. The reroute strategy allows an earlier routed cell track to be reset. The cell trajectory terminates in three conditions. The first condition is when it reaches the last frame of the time-lapse sequence. The second condition is when the score in the next frame is all zeros. The third condition is when the next frame does not have an available cell due to nodes owned by other trajectories, and the merge criteria are not fulfilled.

**FIGURE 7:**
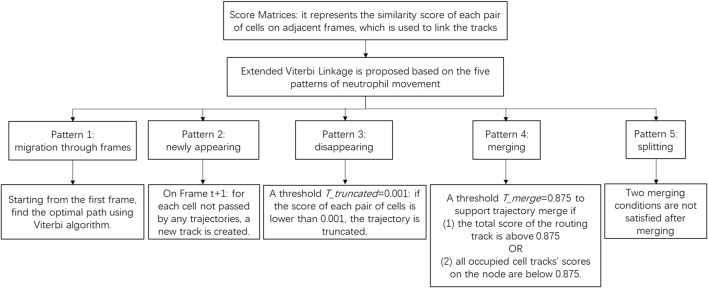
Decision-logic diagram illustrating how merge/split and appearance/disappearance are handled during trajectory building.

In summary, to accommodate varied biological scenarios, $T_{\mathrm{truncated}}$ is used to handle disappearing cells. A newly appearing cell is added if the cell was not passed by any of the trajectories in the next frame. Merging is realized by allowing candidate cells to share more than one trajectory with the threshold $T_{\mathrm{merge}}$ . Splitting occurs when the previously merged candidate cell no longer satisfies merging conditions in subsequent frames. A decision-logic diagram is provided to illustrate how merge/split and appearance/disappearance are handled during trajectory building in Figure 7.

#### 2.4.4 Evaluations for linkage trajectories

After implementing the extended Viterbi algorithm for each time-lapse sequence, all the predicted cell trajectories of each sequence were saved, the same as the ground-truth trajectories. Evaluation metrics were used to measure the performance of the linkage methods. Four measures (Smith et al., 2005) have been selected to evaluate how well a tracker identifies objects, which are defined as follows.

Track purity (*TP*): a measure of the degree to which the predicted tracks ( ∈ ) follow ground-truth objects (*GT*). *TP* is the ratio of frames in which ∈ correctly identifies *GT* to the total number of frames ∈ that exist.

Object purity (*OP*): a measure of the degree to which the ground-truth objects (*GT*) are followed by predicted tracks ( ∈ ). *OP* is the ratio of frames wherein *GT* is correctly identified by ∈ to the total number of frames in which *GT* exists.

Falsely identified tracker (*FIT*): a measure of the degree to which the *GT* is tracked by the incorrect ∈ .

Falsely identified object (*FIO*): a measure of how often ∈ is tracking a different cell than the *GT* it was matched to.

In addition, the average track length was computed.

Average length of tracks: the ratio of the total length of all predicted tracks to the number of predicted tracks.

## 3 Results

We extracted results from each part of the pipeline: cell segmentation, cell motion tracking, and trajectory linkage, respectively.

### 3.1 Segmentation results

A total of 240 ground-truth segmentation images were divided into a train-validation set (192 images) and a test set (48 images) based on the ratio of 8:2. A total of 48 images were used to test the segmentation model as the “unseen” data. The train-validation set was split into a training set (153 images) and a validation set (39 images) in a ratio of 8:2. Table 1 shows the performance of four segmentation models on 48 test images. The U-Net 1 was the structure used in our study. It achieved 0.912 *IoU* and a 0.985 *F1* score, which outperformed the other methods compared. U-Net 2 was the original U-Net model (Ronneberger et al., 2015). The input and output sizes of this model were 572 × 572 and 388 × 388, respectively. Thus, the output masks of this model should be resized to 512 × 512 as the original image size. During this up-sampling resizing process, some mask information was not well preserved. This caused the predicted cells to have a smaller surface area than U-Net 1. It resulted in a lower *IoU* score of 0.697. Some small cells were also missed, which resulted in a high number of *FN*. U-Net 2 had fewer under-segmented cells and more over-segmented cells than U-Net 1 due to its smaller surface area. For the U-Net 3 model, the binary masks were obtained first, and post-processing based on the watershed transform was applied to obtain the instance masks. In the experiments, both the binary masks and the instance masks on the test set were obtained and used to compute the evaluation metrics. The results in Table 1 show that U-Net 3 achieved a lower *IoU* than U-Net 1 but a comparable *F1* score. U-Net 3 (binary mask) achieved a small number of under-segmented and over-segmented cells, but with a slightly higher *FN*, while U-Net 3 (instance mask) achieved higher values of *FN*, under-segmentation, and over-segmentation. The experiments show that the complex instance-based segmentation model does not outperform much compared with a simple U-Net model. Moreover, it took 6–7 times more computation time than U-Net 1. We fine-tuned the parameters of the WMC segmentation algorithm for our neutrophil dataset, which yielded an acceptable accuracy of 0.725 *IoU*, 0.976 *F1* score, and 34 *FN* numbers. In addition, the WMC algorithm has only one under-segmented cell, demonstrating that it performs well in separating the cells that touch. However, it has a relatively high number of 26 over-segmented cells compared with other models and has the lowest efficiency.

**TABLE 1:** Performance of five segmentation models on the test set.

Model	*IoU*	*F1* score	*FN*	Merge	Split	Time(s)
U-Net 1	0.912	0.985	8	18	5	64.00
U-Net 2 (Ronneberger et al., 2015)	0.697	0.956	53	6	7	313.47
U-Net 3 (binary) (Scherr et al., 2020)	0.695	0.984	15	12	4	402.50
U-Net 3 (instance) (Scherr et al., 2020)	0.682	0.985	16	28	12	402.50
Watershed (Yan and Verbeek, 2012)	0.725	0.976	34	1	26	702.58

**FIGURE 8:**
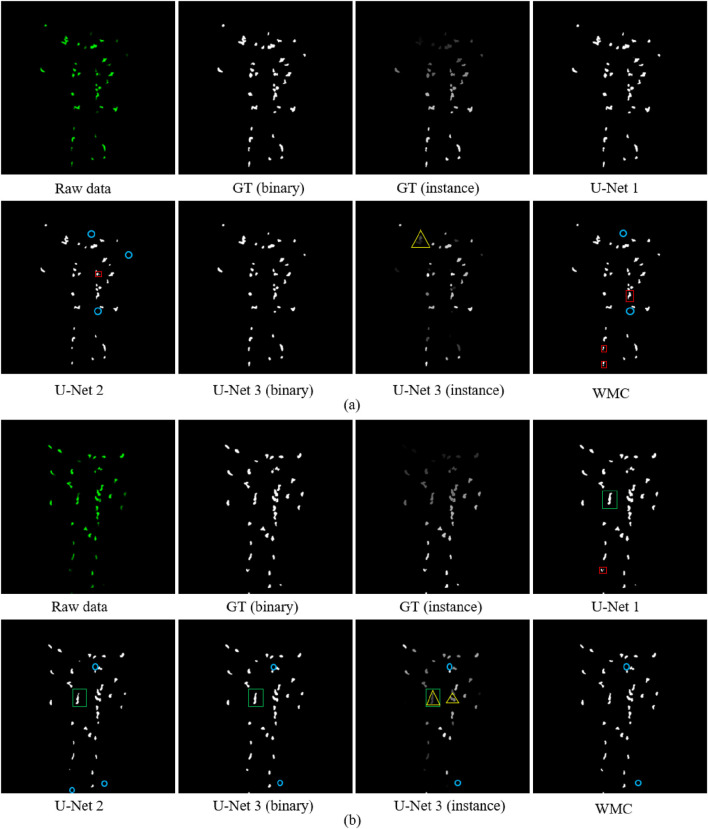
**(a,b)** Two examples of segmentation results. The examples show the raw data and the ground truth of the binary mask and the instance mask, respectively. The five segmentation results (U-Net 1, U-Net 2, U-Net 3 (binary), U-Net 3 (instance), and watershed) are shown. The red rectangular boxes represent the over-segmentation samples. The green rectangles represent the under-segmentation samples. The blue circles represent *FN*. The yellow triangles represent the samples that are expected to be separated by the instance segmentation model; however, the model does not yet work very well.

Figure 8 shows the two examples of the different segmentation results. U-Net 1 obtained the closest segmentation results to the ground truth. The surface area of the predicted cells in U-Net 2 is much smaller, which leads to more *FN* and over-segmented samples. In Figure 8, this is indicated by blue circles and red rectangles. The WMC algorithm also resulted in more *FN* and over-segmented objects. This corresponds to the results presented in Table 1. U-Net 3 (binary) was the most comparable model to U-Net 1; however, it had a lower computational efficiency. U-Net 3 (instance) was expected to separate the touched cells. This worked well in some cases. However, it sometimes failed, as shown with the yellow triangles in Figure 8.

All these segmentation results have advantages and disadvantages. We selected the one that performed better using less computation time for the cell-tracking task in the next step. Based on the analysis above, U-Net 1 was selected as the segmentation model in our pipeline.

### 3.2 Tracking results

To predict the most possible location of each candidate cell on the test set, three models, including a pretrained model, a model built from scratch, and a fine-tuned model, were trained. The predicted candidate cell was matched to both the original image and segmentation mask to determine whether they matched the corresponding region of interest (ROI) of the cell. If so, it was counted as a correct prediction; otherwise, it was counted as a wrong prediction. In this process, the original image helps provide the intensity value, and the segmentation mask provides the location of the cell. Our ground-truth trajectories have no splitting annotations; the models were expected to predict only one cell in each output image. If there is more than one cell ROI predicted from the output of the model, the ROI with the highest average intensity is selected as the final localized cell. Subsequently, the total number of cells (predictions) on each of the ground-truth trajectories in the test set was also counted. The error rate is calculated as the number of erroneous predictions divided by the total number of predictions.

Table 2 shows the error rate of the three models. Among the 11,614 predictions, 572 cells, 781 cells, and 578 cells were mis-predicted by the from-scratch model, the pretrained model, and the fine-tuned model, respectively. From Table 2, the pretrained model produced the highest error rate of 0.067. This seems to be reasonable as this model was trained on an *E. coli* cells dataset, which is a cell type totally different from neutrophils. The from-scratch model and the fine-tuned model obtained a comparable error rate, with the from-scratch model having the lowest one.

**TABLE 2:** Predicted errors of each tracking model on the test set.

Models	Number of erroneous predictions	Number of predictions	Error rate
From-scratch model	572	11,641	0.049
Pretrained model	781	11,641	0.067
Fine-tuned model	578	11,641	0.050

In this study, we selected the outputs of the fine-tuned model to perform the next step of cell linkage rather than those of the from-scratch model because we selected the best ROI as the candidate cell in the prediction frame to compare it with the ground-truth frame during error rate calculation. If one frame has multiple cells, which is often observed in the output of the model trained from scratch, the remaining cells are not counted in the evaluation. Subsequently, the error rate does not reflect the downstream trajectory error, as we observed from the trajectories obtained in the output of the from-scratch model. We conducted an experiment to calculate and compare the trajectory errors. The evaluation metrics, *FIT*, *FIO*, *TP*, and *OP*, of the four linkage methods based on the output of the from-scratch model and the fine-tuned model are presented in Supplementary Section 1. The results confirmed that the fine-tuned model is a better option for cell linkage.

### 3.3 Linkage results

For a 120-frame time-lapse sequence, a total of 119 score matrices were obtained. The score was calculated based on the predictions of the tracking model and was defined as the overlapping area between the predicted cell on the current frame and each cell on the previous frame. Based on the score matrices, we performed our extended Viterbi algorithm, as well as several different linkage methods, to compare the performance. The first one is the DeLTA linkage method (Lugagne et al., 2020). DeLTA linkage is a straightforward method to link trajectories. It starts from every cell in the first frame to find the candidate cell in the next frame with the highest score value. If the values of two candidate cells are the same, it links the first candidate cell directly, while the other cell can start a new trajectory. Each cell node can only be passed once by one trajectory. The second involved linkage method is the Hungarian algorithm (Atsuki et al., 2015). The Hungarian algorithm aims to find the optimal path in the score matrices by solving an assignment problem. For the linkage process, it starts from each cell in the first frame to link the candidate cell frame by frame, until no candidate cell can be linked. The end condition depends on the configured threshold. Each frame was looped, and the unpassed cell was treated as the start of a new trajectory.

Comparing the two linkage methods, the Viterbi algorithm is more powerful due to its concept of optimization of a global linkage. Viterbi optimizes the path by taking the relationship of all frames into account, not only the adjacent frames. A basic Viterbi algorithm was also compared to show the improvement of our extended Viterbi algorithm. In addition, a rule-based tracking algorithm named KDE with mean shift (Yan et al., 2009) was used to demonstrate the outperformance of our pipeline, incorporating a deep-learning-based tracking model and an extended Viterbi linkage method. In the experiment, the threshold $T_{\mathrm{truncated}}$ was set to 0.001 and $T_{\mathrm{merge}}$ was set to 0.875.

**FIGURE 9:**
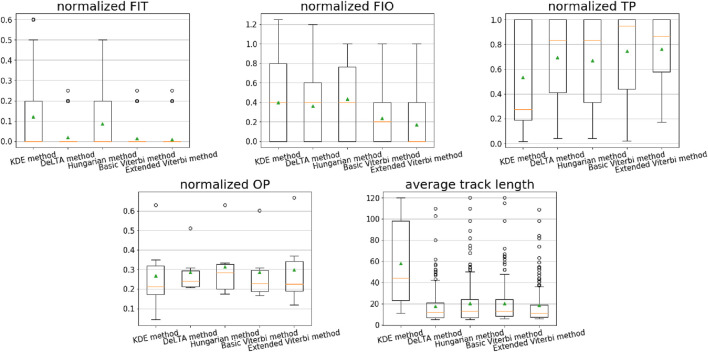
Performance of different linkage methods with the selected evaluation metrics on the test set. The metrics are falsely identified track (*FIT*), falsely identified object (*FIO*), track purity (*TP*), object purity (*OP*), and average predicted track length. In the boxplots, the green triangles show the mean values, the orange lines are the median values, the boxes represent the first to third quartiles of the data, the whiskers indicate the lower and upper extremes (1.5 times the interquartile range), and the black circles are the outliers.

Figure 9 shows the performance comparison of the methods in our evaluation. Figure 9 shows the box plots of the evaluation metrics determined for the different linkage methods applied to the test set. The evaluation metrics *FIT*, *FIO*, *TP*, and *OP* are normalized. In addition, a statistical analysis was performed using the Kruskal–Wallis test, followed by Dunn’s post-hoc test to assess the significance of the differences in performance. Specifically, we first performed the Kruskal–Wallis test. A *p*-value <0.05 indicated significant differences among the five methods. Subsequently, Dunn’s post-hoc test was conducted for pairwise comparisons, with the Benjamini–Hochberg (FDR) correction applied for multiple testing. Moreover, the average track length is computed. The normalized values represent the average number of errors (*FIT* and *FIO*) and purities (*TP* and *OP*) per frame per cell on the test set from the ground truth. In the box plots (cf. Figure 9), the green triangles represent the mean value of each evaluation. The resulting values are presented in Table 3; these mean values correspond to Figure 9. From Table 3, our extended Viterbi achieved the lowest mean value of 0.010 *FIT* and 0.173 *FIO* of the methods in our comparison. For purity evaluations, the extended Viterbi also yielded the highest *TP* value of 0.763 as well as a second-best *OP* of 0.299. For statistical analysis, our extended Viterbi method shows a significant difference in *FIO* compared with the other four methods. From the experiments, our extended Viterbi algorithm produces a better cell-tracking result.

**TABLE 3:** Performance of each linkage method. The footnote is the rank for all compared methods of each evaluation metric.

Algorithm	Normalized *FIT*	Normalized *FIO*	Normalized *TP*	Normalized *OP*	Average track length
KDE (Yan et al., 2009)	0.122	0.399	0.537	0.268	57.844
DeLTA (Lugagne et al., 2020)	0.020	0.365	0.694	0.285	17.520
Hungarian (Atsuki et al., 2015)	0.087	0.437	0.668	0.314	20.356
Basic Viterbi	0.015	0.237	0.747	0.286	20.158
Extended Viterbi	$0.010^{1}$	$0.173^{1}$	$0.763^{1}$	$0.299^{2}$	$18.427^{4}$

The results in Figure 9 show that compared with a rule-based KDE method, a deep learning-based tracking model, together with any of the aforementioned linkage methods, performs much better for our cell-tracking task. The KDE method obtained the highest *FIT* and the lowest *TP* and *OP*. It only outperformed the Hungarian algorithm by 0.01 *FIO*. This is due to the features used in the KDE method; that is, the kernel density of each cell. Only using kernel density is limited to distinguishing the cell tracks on the neutrophil dataset because of the complex morphology and migration behavior of neutrophils. However, a deep learning model can learn more features of cell morphology and movement from the ground-truth trajectories. In Figure 9, the DeLTA, Hungarian, basic Viterbi, and extended Viterbi methods show that they achieve a comparable average track length. The KDE algorithm creates a much longer average length than the other four methods. This is because it does not solve the merge/split problems. Once the cells are merged, the trajectories of these cells overlap. It causes a longer track length of each cell trajectory, which leads to a longer average track length. The original Viterbi algorithm exhibits the same problem. We applied a post-processing for the basic Viterbi algorithm. The trajectory with a lower score than the previous cell on the previous frame was truncated. The post-processing is helpful and improves the performance.

**FIGURE 10:**
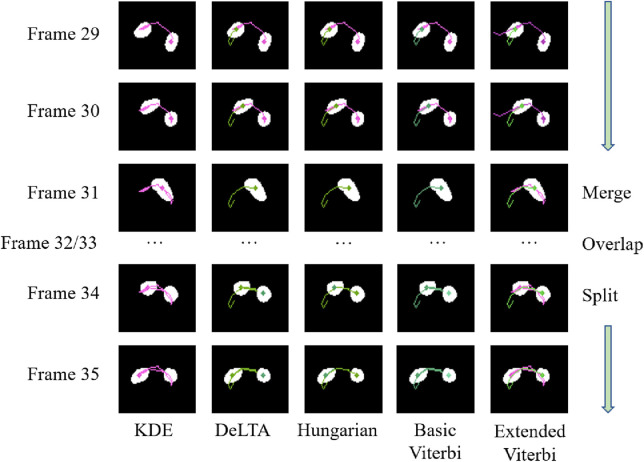
Comparison of how these methods solve the cell merge/split problem.

We designed different strategies for our extended Viterbi algorithm to deal with the merge/split problems. The experiments show that the extended Viterbi algorithm further increases the performance. A case of how the merge/split problem is solved by the five algorithms is depicted in Figure 10. We present a series of consecutive frames between frames 29 and 35 of one particular time-lapse sequence. For the KDE algorithm, only the right cell, represented with a purple line, was tracked; the left cell was missed. For the DeLTA algorithm, the left cell, represented with a green line, was linked to the left one when the cells split in frame 34. This is not an expected direction. The right cell was treated as the start of a new cell trajectory. The same situation occurred with the basic Viterbi algorithm. For the Hungarian algorithm, the left cell, represented with a green line, was linked to the right cell after the split, which is the expected cell moving direction. For our extended Viterbi algorithm, the merge/split problem was solved. The left cell, represented with the green line, is expected to move from left to right even after a collision, whereas the right cell, represented with the purple line, moves from right to left.

### 3.4 Measurements of neutrophil migration properties

After evaluating the performance of each step in the cell-tracking pipeline, we quantified several standard neutrophil migration properties to assess how different tracking methods affect the inferred migration behavior. From our previous work (Hu et al., 2021), biologists measured net displacement, meandering index, and mean speed using manual tracking data. Net displacement is calculated as the Euclidean distance between the cell’s positions in the first and last frames. The meandering index is defined as the net distance divided by the total distance traveled by the cell. Mean speed is defined as the total distance traveled divided by the travel time. These three measurements are used to observe the effect of myd88 mutation on distant neutrophil migration behavior. In this experiment, there are six 3-dpf zebrafish larvae in the wild-type control group and five zebrafish larvae in the myd88 mutation group. A distant neutrophil is defined as a cell that travels far from the wound site. Cells with a starting point of movements localized further than 200 µm from the wound site were classified as distant neutrophils. The results revealed that the myd88 mutation did not affect the mean speed of distant neutrophils upon wounding. However, a significant decrease in net displacement and meandering index was consistently observed in the myd88 mutant group.

**FIGURE 11:**
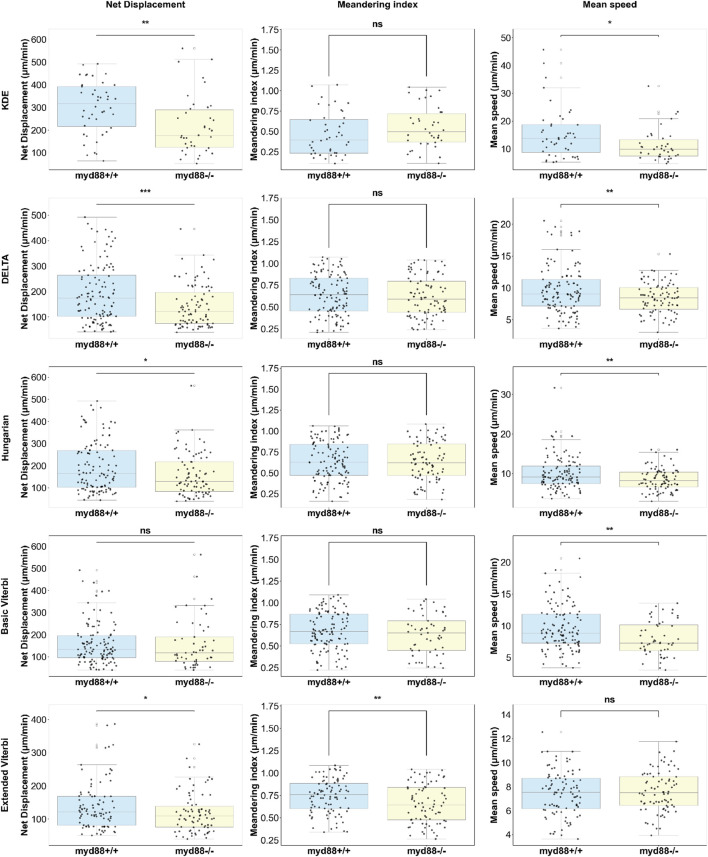
Comparison of neutrophil migration properties with different linkage methods. An independent samples t-test was used to assess significance (ns, non-significance, ∗ *p* <0.05 , ∗∗ *p* <0.01 , and ∗∗∗ *p* <0.001 ), and data are shown as the mean ± SD.

In this study, we used the same 2-h time-lapse sequences for the evaluation. After cell tracking, we derived 487 tracks from the control group and 342 tracks from the myd88 mutation group. Due to a lack of wound site information, distant neutrophils were defined based on average net displacement between frames longer than 2.4 µm. In the previous work (Hu et al., 2021), we observed that net displacement and meandering index are significantly decreased in the local resident neutrophil groups compared to the distant neutrophil groups. Thus, average net displacement was chosen to obtain cells with longer traveling distance and to correspond to the previous setup of 200 µm. We compared results from different linkage methods in Figure 11. The results demonstrated that the extended Viterbi algorithm was the only method that showed the same trend for all three measurements. Note that even after adjusting the parameter of the average net displacement, the other methods did not show the same trend. The extended Viterbi algorithm, however, showed a consistent trend within 2.2–2.5 µm, as shown in Supplementary Figure S1.

### 3.5 Results on other datasets

To prove the robustness of our pipeline, two more experiments were conducted on datasets of two cell types with the same threshold setup. One dataset is downloaded from the cell-tracking challenge (Maška et al., 2014). It contains the stained nuclei of HL60 cells (Fluo_N2DH_SIM) and the HeLa cells stably expressing H2b-GFP (Fluo_N2DL_HeLa). The cells in this dataset move slowly and exhibit only mitotic behavior. The other type of data is MTLn3 plGFP cells (Yan et al., 2009; Yan and Verbeek, 2012). This dataset consists of two groups. One is the experiment group, in which the cells move slowly without mitosis or any other behavior. The other is the control group, in which the cells move faster but do not undergo mitosis or any other behavior.

Both datasets have simpler cell behavior and slower movement than neutrophils. The length of the time-lapse sequence is much shorter than the 120-frame neutrophil sequences, which facilitates the cell-tracking task. The HL60 dataset has five time-lapse sequences in total, with 65 frames per time-lapse sequence. The cell density is 10–40 cells per frame, which is comparable to the neutrophil dataset. The MTLn3 dataset has two time-lapse sequences with 30 frames per sequence but with a very high cell density of approximately 80–100 cells per frame.

**FIGURE 12:**
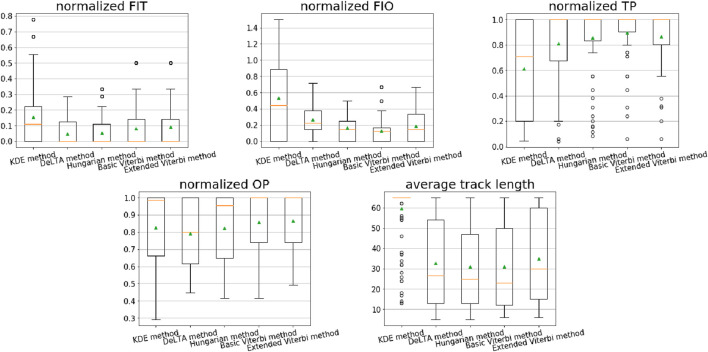
Performance of different linkage methods with the selected evaluation metrics on the HL60/HeLa dataset. The metrics are *FIT*, *FIO*, *TP*, *OP*, and average predicted track length. In the box plots, the green triangles show the mean values, the orange lines are the median values, the boxes represent the first to third quartiles of the data, the whiskers indicate the lower and upper extremes (1.5 times the interquartile range), and the black circles are the outliers.

**FIGURE 13:**
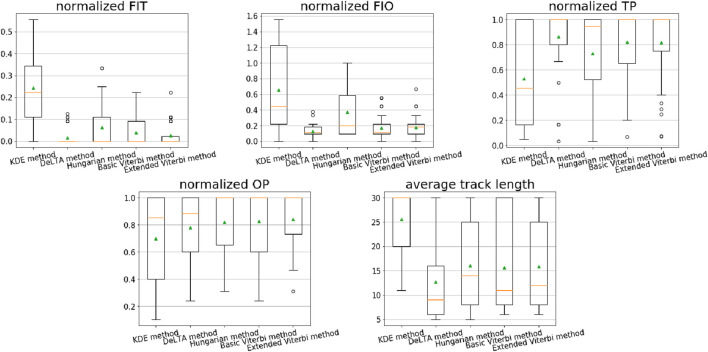
Performance of different linkage methods with the selected evaluation metrics on the MTLn3 dataset. The metrics are *FIT*, *FIO*, *TP*, *OP*, and average predicted track length. In the box plots, the green triangles show the mean values, the orange lines are the median value, the boxes represent the first to third quartiles of the data, the whiskers indicate the lower and upper extremes (1.5 times the interquartile range), and the black circles are the outliers.

We retrained the U-Net 1 segmentation model and tracking model on the two new datasets, respectively. Subsequently, the same linkage methods were implemented. The performance results are shown in Figures 12, 13. Tables 4, 5 present the mean values (green triangles) corresponding to Figures 12, 13. A statistical analysis was performed using the Kruskal–Wallis test, followed by Dunn’s post-hoc test to assess the significance of the differences in performance.

**TABLE 4:** Performance of each tracking model on the HL60/HeLa dataset. The footnote shows the rank of each evaluation metric.

Algorithm	Normalized *FIT*	Normalized *FIO*	Normalized *TP*	Normalized *OP*	Average track length
KDE (Yan et al., 2009)	$0.154^{5}$	$0.537^{5}$	$0.612^{5}$	$0.825^{3}$	$59.801^{1}$
DeLTA (Lugagne et al., 2020)	$0.048^{1}$	$0.265^{4}$	$0.808^{4}$	$0.790^{5}$	$32.577^{3}$
Hungarian (Atsuki et al., 2015)	$0.053^{2}$	$0.165^{2}$	$0.857^{3}$	$0.823^{4}$	$30.867^{5}$
Basic Viterbi	$0.082^{3}$	$0.125^{1}$	$0.897^{1}$	$0.856^{2}$	$30.915^{4}$
Extended Viterbi	$0.091^{4}$	$0.186^{3}$	$0.864^{2}$	$0.866^{1}$	$34.866^{2}$

**TABLE 5:** Performance of each tracking model on the MTLn3 dataset. The footnote shows the rank of each evaluation metric.

Algorithm	Normalized *FIT*	Normalized *FIO*	Normalized *TP*	Normalized *OP*	Average track length
KDE (Yan et al., 2009)	$0.243^{5}$	$0.658^{5}$	$0.532^{5}$	$0.700^{5}$	$25.509^{1}$
DeLTA (Lugagne et al., 2020)	$0.015^{1}$	$0.126^{1}$	$0.862^{1}$	$0.779^{4}$	$12.670^{5}$
Hungarian (Atsuki et al., 2015)	$0.062^{4}$	$0.373^{4}$	$0.732^{4}$	$0.819^{3}$	$16.010^{2}$
Basic Viterbi	$0.040^{3}$	$0.172^{2}$	$0.820^{2}$	$0.827^{2}$	$15.650^{4}$
Extended Viterbi	$0.028^{2}$	$0.175^{3}$	$0.818^{3}$	$0.841^{1}$	$15.796^{3}$

For the HL60/HeLa dataset, the basic Viterbi algorithm achieved the best score in *FIO* and *TP* and a second-best score of *OP* among these different methods. The extended Viterbi only achieved the best performance of *OP*. This experiment demonstrated that our strategies work better on a cell dataset with more complex patterns, such as neutrophils. For the cell dataset with simple patterns, the basic Viterbi is robust and outperforms the others. Compared with the KDE method, deep learning methods still perform better than a machine learning-based method, which is consistent with the results on neutrophils. In statistical analysis, both basic Viterbi and extended Viterbi show significant differences in *FIT* compared to the other three methods. Basic Viterbi showed a significant difference in *FIO* compared to the other four methods.

For the MTLn3 dataset, the performance of the two Viterbi-based algorithms is comparable with the DeLTA and Hungarian methods. These four methods have their advantages and disadvantages but still perform better than the KDE method. The extended Viterbi achieved the best score of *OP*, whereas DeLTA obtained the best *FIT*, *FIO*, and *TP*. The basic Viterbi yielded the second-best *FIO*, *TP*, and *OP*. However, there is no statistically significant difference in *FIO* between the DeLTA, basic Viterbi, and extended Viterbi methods. Possible reasons that the Viterbi-based algorithms are not outperformed by the other two methods are as follows: (1) the experiment contains only two time-lapse sequences with 30 frames per sequence. The number of data points is rather limited for good training of a deep learning model. (2) Furthermore, the high cell density of the sequences easily causes linkage errors due to the global linkage method of the Viterbi algorithm. This is because the cells are very close to each other. The prediction would result in an overlap of two or more comparable adjacent cells, causing about the same score in the matrix. It easily leads to an error trajectory in the global linkage process. Therefore, Viterbi-based algorithms are more robust on time-lapse sequences with a low or medium cell density rather than a very high density.

### 3.6 Comparison with an existing cell-tracking tool

Additionally, we compared our pipeline with the existing cell-tracking tool: TrackMate (Tinevez et al., 2017). TrackMate works on cell tracking in two separate steps: cell detection and particle-linkage. For particle-linkage, a linear assignment problem (LAP) is proposed. To solve this LAP during the implementation, the tool relies on the Munkres & Kuhn (Hungarian) algorithm. Our study incorporated the classic Hungarian algorithm for the comparison of linkage algorithms. Thus, we indirectly compared our method with TrackMate regarding the linkage strategies. Furthermore, we compared the result of TrackMate V8 in FIJI with the result of the extended Viterbi algorithm, focusing on merging and splitting events via visual inspection. In TrackMate, we used the LoG detector with a blob diameter of 25 µm. Auto initial thresholding was used for filters on spots. In the LAP tracker, we set a max distance to 50 µm. We conducted experiments both with and without merging/splitting options. Representative screenshots of the results can be found in Supplementary Section 2. The results confirmed that our extended Viterbi algorithm is improved when solving merging and splitting problems and can maintain the lineage information of cells over time compared to TrackMate.

## 4 Discussion

In this article, we developed a pipeline incorporating cell segmentation, cell motion tracking between two frames, and trajectory linkage to track the complex migration patterns of neutrophils in the time-lapse sequences. The pipeline integrates two U-Net models to segment cells and learn the cell movement behavior, respectively. Cell segmentation is an important step in the whole pipeline. A lower-performing segmentation model could further influence the performance of trajectory linkage. Several other cell segmentation tools, such as Cellpose (Stringer et al., 2020) or CellSAM (Marks et al., 2025), provide pretrained/foundation models for a range of cell types. In the case of Cellpose, these pretrained models can be further fine-tuned for specific applications, potentially including neutrophil segmentation in zebrafish, with relatively modest computational cost. In the future, we will explore a better segmentation model for neutrophils.

The extended Viterbi algorithm can handle different patterns in cell migration, such as appearing, disappearing, and going in or out of the field of view, to more accurately link the cell trajectories. Compared with representative linkage methods in cell tracking, our algorithm achieved superior performance and provides an effective approach to help understand cell migration behavior. In addition, our algorithm can reproduce the same trends of neutrophil migration properties observed with manual annotation. In future work, we will extend our work to 3D cell tracking using our neutrophil dataset. 3D data provide more spatial information in the Z-axis and are expected to track the cell trajectories in a better way.

In addition, we compared our pipeline with the existing cell-tracking tool: TrackMate. The results demonstrated that our extended Viterbi algorithm offers an improved method of solving merging and splitting problems compared to TrackMate.

In conclusion, neutrophil tracking is an example of challenging tracking tasks due to the complexity of cell morphology and fast-moving dynamics. It also demonstrates complex tracking challenges caused by unavoidable undersampling during microscopy imaging due to a large scan area and measurement design constraints. Through systematic comparisons across multiple detection methods, cell linkage algorithms, datasets, and existing cell-tracking tools, our pipeline shows robust and improved performance in tracking highly motile cells under challenging conditions, including merging/splitting and constrained spatiotemporal imaging settings.

## Data Availability

The raw data supporting the conclusions of this article will be made available by the authors, without undue reservation.
