# Supplementary material for: An automated cell-tracking pipeline for the analysis of neutrophil dynamics
Source: Front Bioinform. 2026 Apr 21;6:1748364. doi: 10.3389/fbinf.2026.1748364 (PMC13139118; doi:10.3389/fbinf.2026.1748364)
Supplement: Supplementary file 1 [file DataSheet1.pdf]

Supplementary Material

1 COMPARISON BETWEEN FROM-SCRATCH MODEL AND FINE-TUNED MODEL FOR TRACKING

In order to compare the trajectory errors, we did experiment to calculate the evaluation metrics *FIT*, *FIO*, *TP*, *OP* of four linkage methods based on the output of from-scratch model in Figure S1 and Table S1 corresponding to the result of fine-tuned model in Table 3. The results showed that, for DeLTA method, all of these four metrics derived from model from scratch are not better than fine-tuned model in Table 3. For Hungarian method, *FIT* and *OP* from model from scratch are better than fine-tuned model, the other two are not. From-scratch model has the highest performance impact for Basic Viterbi, where *FIO* is drastically increased from 0.237 to 11.3. Contrary, *OP* from model from scratch is better than fine-tuned model. For extended Viterbi, only *TP* from model from scratch is better than fine-tuned model, the other three are not. Potential reason is that Delta linkage and Hungarian linkage enforce hard one-to-one matching per frame. These strict rules help alleviate the impact of having more than one candidate cells. The advantage of Basic Viterbi method is its emphasis on global path optimization. However, it is sensitive to falsely predicted candidate cells and over-segmentation. It treats all candidate cells as valid hidden states and lacks the strict one-to-one assignment constraint between frames. Our extended Viterbi, however, imposed stricter constraint when dealing with merge/split events between frames. Thus, the impact to the extended Viterbi linkage method is less than basic Viterbi method. In summary, the results showed that from-scratch model does not perform better than fine-tuned model.

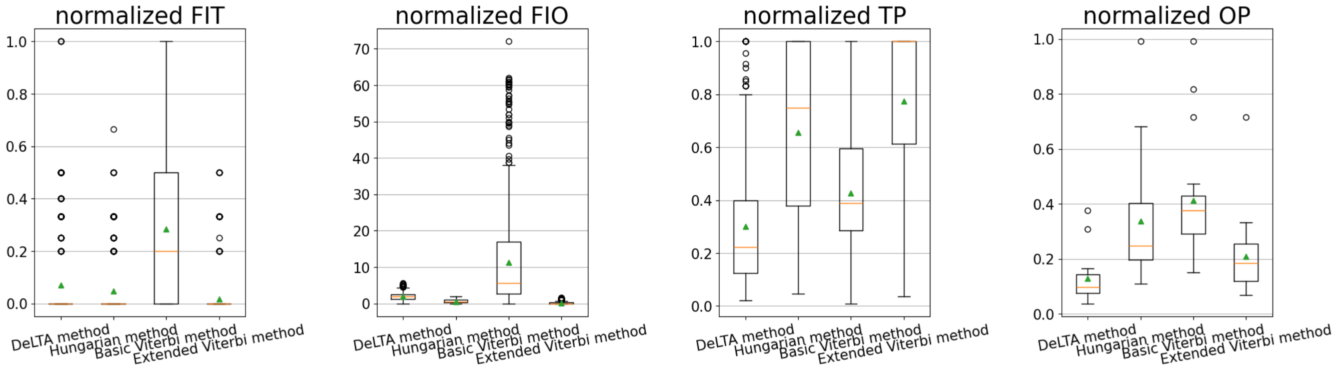

Figure S1. The comparison results of different linkage methods based on the from-scratch model.

Table S1. The performance of four linkage methods based on the from-scratch model.

Algorithms	Normalized *FIT*	Normalized *FIO*	Normalized *TP*	Normalized *OP*
DeLTA	0.070	1.960	0.301	0.127
Hungarian	0.048	0.550	0.657	0.337
Basic Viterbi	0.285	11.300	0.428	0.412
Extended Viterbi	0.017	0.189	0.775	0.21

2 MEASUREMENTS OF NEUTROPHIL MIGRATION PROPERTIES

In this study, we used the same 2-hour time-lapse sequences for the evaluation. After cell tracking, we derived 487 tracks from control group and 342 tracks from myd88 mutation group. We compared results of different linkage methods with varied average net displacement.

	KDE			DELTA			Hungarian			Basic Viterbi			Extended Viterbi		
Distance per frame (μm)	Net Displacement	Meandering index	Mean speed	Net Displacement	Meandering index	Mean speed	Net Displacement	Meandering index	Mean speed	Net Displacement	Meandering index	Mean speed	Net Displacement	Meandering index	Mean speed
1	***	ns	ns	***	*	*	*	ns	ns	ns	ns	**	ns	ns	ns
1,1	***	ns	ns	**	ns	*	*	ns	ns	ns	ns	**	*	*	ns
1,2	***	ns	ns	**	ns	ns	*	ns	ns	ns	ns	**	*	*	ns
1,3	***	ns	ns	**	ns	*	**	ns	*	ns	ns	**	*	*	ns
1,4	***	ns	ns	**	ns	*	**	ns	*	ns	*	**	ns	*	ns
1,5	***	ns	ns	**	ns	*	**	ns	*	ns	ns	**	ns	*	ns
1,6	**	ns	ns	**	ns	ns	**	ns	ns	ns	ns	**	ns	*	ns
1,7	**	ns	ns	*	ns	*	**	ns	ns	ns	*	**	ns	ns	ns
1,8	**	ns	ns	*	ns	*	**	ns	ns	ns	ns	**	ns	ns	ns
1,9	***	ns	*	**	ns	*	**	ns	*	ns	ns	**	ns	ns	ns
2	***	ns	*	*	ns	*	**	ns	*	ns	ns	**	ns	ns	ns
2,1	***	ns	*	*	ns	*	**	ns	*	ns	ns	**	ns	ns	ns
2,2	***	ns	*	*	ns	*	**	ns	*	ns	ns	**	*	*	ns
2,3	***	ns	**	**	ns	**	**	ns	*	ns	ns	**	*	*	ns
2,4	***	ns	**	*	ns	**	**	ns	*	ns	ns	**	*	**	ns
2,5	**	ns	**	*	ns	**	**	ns	*	ns	ns	**	*	*	ns
2,6	**	ns	**	*	ns	**	**	ns	*	ns	ns	**	ns	*	ns
2,7	**	ns	**	*	ns	**	**	ns	*	ns	ns	**	ns	ns	ns
2,8	**	ns	**	ns	ns	**	*	ns	*	ns	ns	**	ns	ns	ns
2,9	***	ns	**	*	ns	**	*	ns	*	ns	ns	**	ns	ns	ns

Figure S2. Results of different linkage methods with varied average net displacement. An independent samples t-test was used to assess significance (ns, non-significance, $*P < 0.05$, $**P < 0.01$, $***P < 0.001$)

3 COMPARISON WITH TRACKMATE

We did experiment with the TrackMate V8 in FIJI. We used LAP tracker to track neutrophil and we aimed to compare the tracking result of merging/splitting via visual inspection.
The first case is from frame 19 to frame 27, it is the first time the two cells on the right up corner merged. The result of LAP tracker was shown in Figure 5. The result of extended Viterbi was shown in Figure 6. Both algorithms found this merging situation challenging. In Figure 5, at frame 24, when two cells were splitting, the left cell in the red bounding box lost its trajectory. We checked the step of LoG detection and this cell was detected. The reason could be that LAP does not deal with splitting and merging situations well. It treats two cells as one when they merge and new cell is created when two cells split. In Figure 6, extended Viterbi algorithm managed to treat two cells as two when they merge. At frame 24, after splitting, it missed one cell on the left in the red bounding box, but it managed to create a new track for this cell. In this way, it handles the cells which are easily missed during merging and splitting.

The second case is from frame 30 to frame 35, the result of LAP tracker was shown in Figure 7. The result of extended Viterbi was shown in Figure 8. In Figure 7, at frame 30, the two cells were going to merge again. From frame 31 to 33, they were merged. When they were splitting at frame 34, one cell went to left with red track. The other cell started a new track. We observed that LAP algorithm does not handle the track of a cell when it constantly encounters merging and splitting situations. But once the cell stops merging and splitting, LAP algorithm is able to create a new track. In Figure 8, our Extended Viterbi algorithm solved it well. The green track (left cell) goes to right direction and the purple track (right cell) goes to left after splitting. They kept the same movement directions.

In addition, we tested using merging/splitting configurations in TrackMate. The tracking result is shown in Figure S7. We observed that, when the two cells were merging in frames 20-23 and splitting at

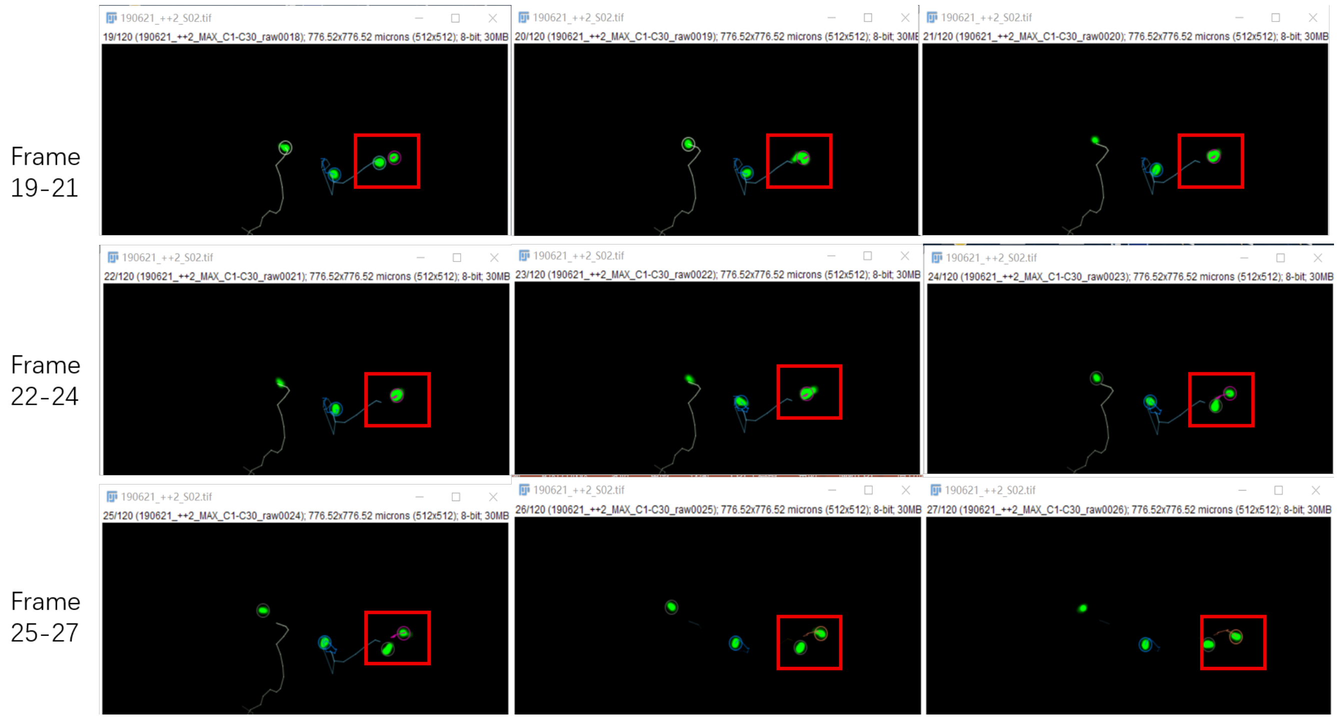

Figure S3. The result of LAP tracker in Case 1.

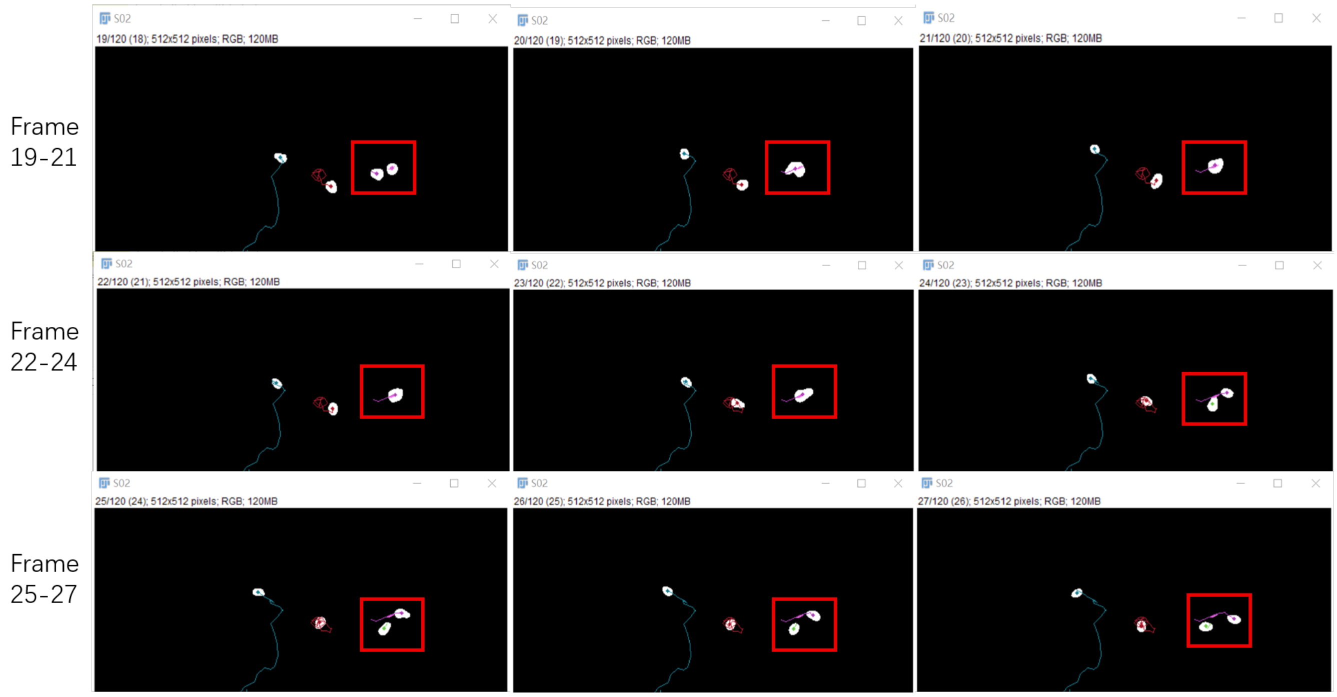

Figure S4. The result of Extended Viterbi algorithm in Case 1.

frame 24, the trajectories are connected well but with the same colored track. The cells were grouped to the same trajectory when the merging/splitting events happened. The potential explanation is that, the merging/splitting handling for LAP algorithm only establishes the spatial–temporal topological connectivity between cells. The handling is not able to assign persistent individual identities to cells and maintain their

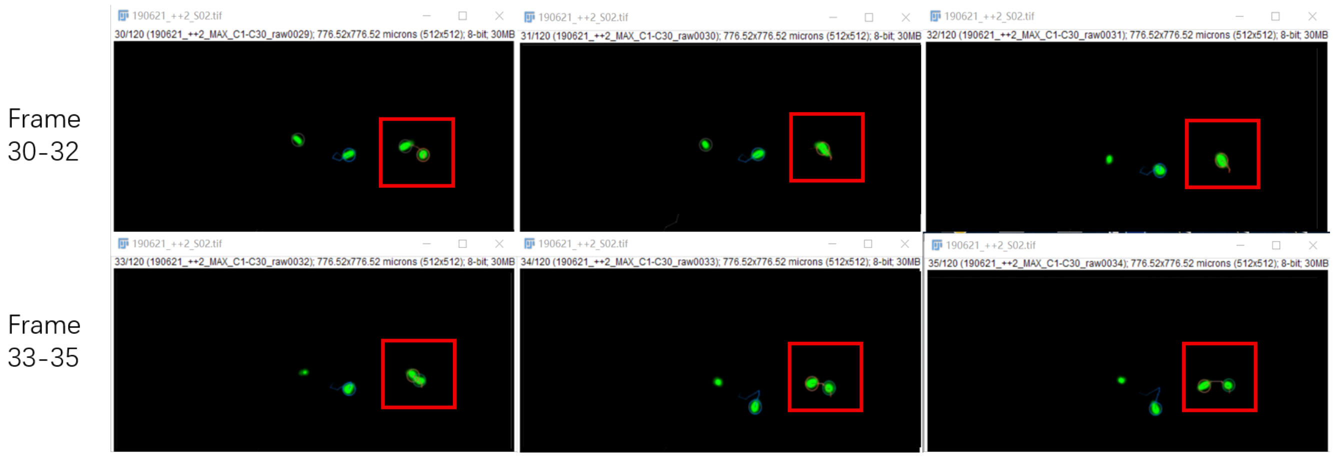

Figure S5. The result of LAP tracker in Case 2.

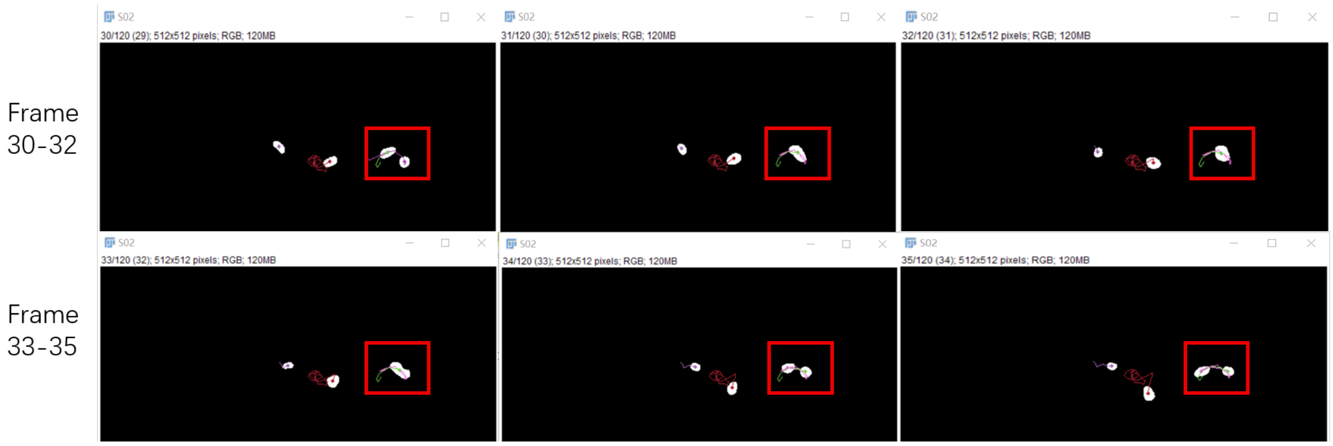

Figure S6. The result of Extended Viterbi algorithm in Case 2.

lineage information over time. Consequently, the unique cell identity is lost during tracking. Figure 3 shows the tracks in TrackScheme. As long as the cells merged, they were all connected into a single track based on a topological graph, without individual identification. However, our extended Viterbi method takes the cell identity into account and maintains the lineage information of cells over time.

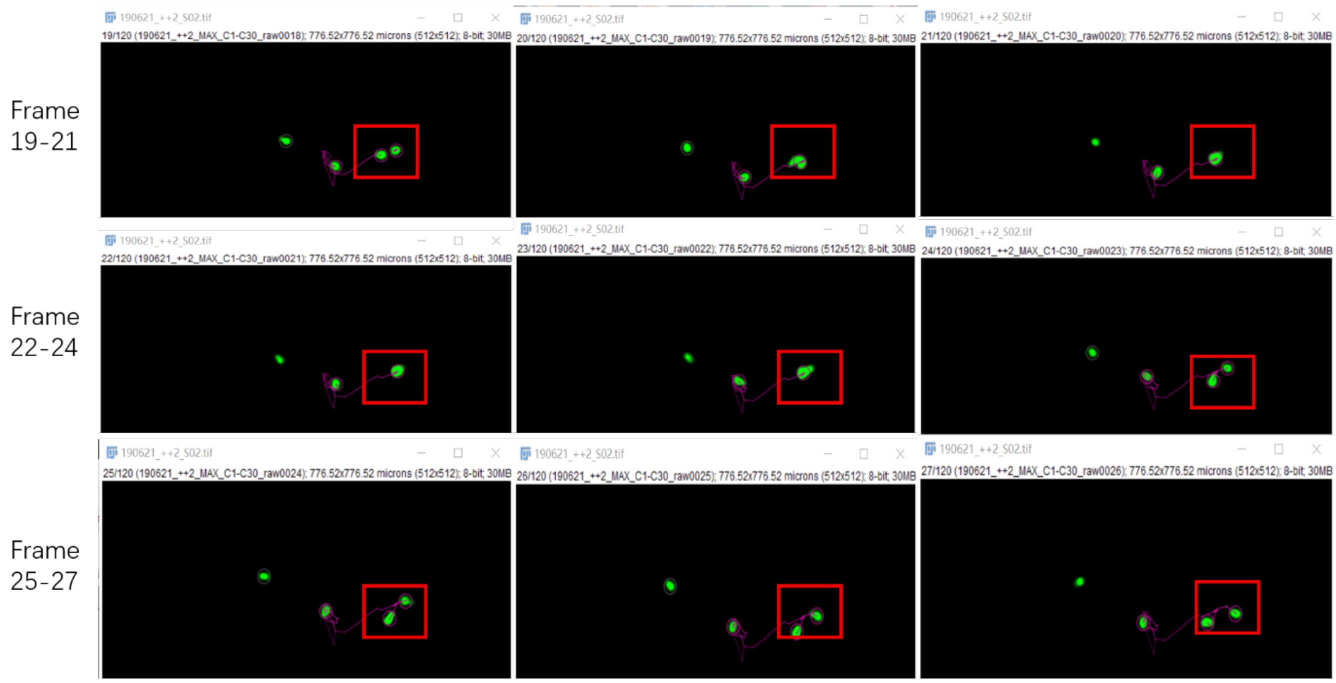

Figure S7. The result of LAP tracker with merging/splitting configuration in Case 1.

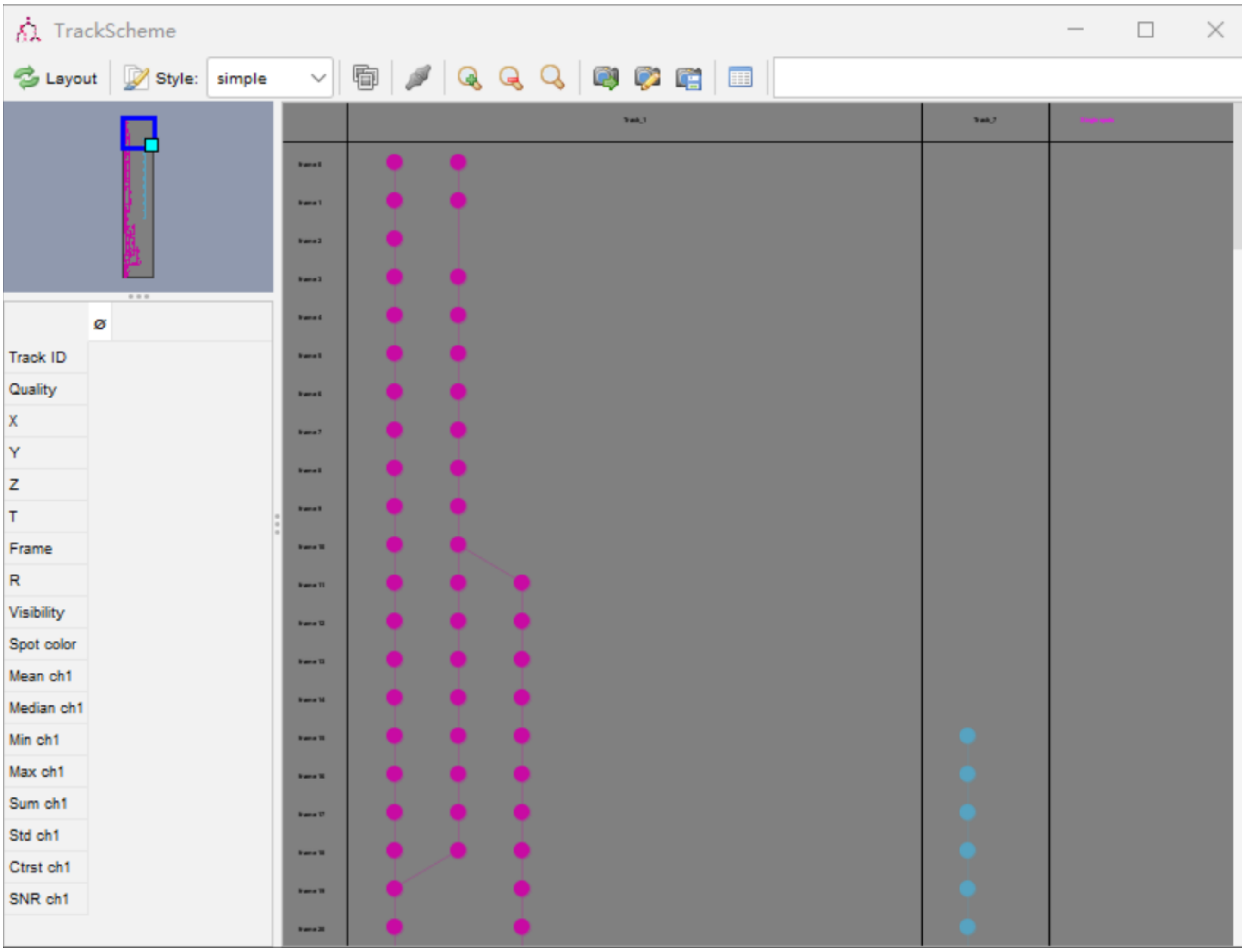

Figure S8. The result of tracks in TrackScheme.
